# IVIg protects the 3xTg-AD mouse model of Alzheimer’s disease from memory deficit and Aβ pathology

**DOI:** 10.1186/1742-2094-11-54

**Published:** 2014-03-22

**Authors:** Isabelle St-Amour, Isabelle Paré, Cyntia Tremblay, Katherine Coulombe, Renée Bazin, Frédéric Calon

**Affiliations:** 1 Centre de Recherche du CHU de Québec, 2705, Boulevard Laurier, Québec, QC G1V 4G2, Canada; 2 Faculté de pharmacie, 1050, Avenue de la Médecine, Université Laval, Québec, QC G1V 0A6, Canada; 3 Département de Recherche et Développement, Héma-Québec, 1070, Avenue des Sciences-de-la-Vie, Québec, QC G1V 5C3, Canada

**Keywords:** Alzheimer’s disease, Intravenous immunoglobulin, Aβ oligomers, 3xTg-AD, Fractalkine, Immunization, CX3CR1

## Abstract

**Background:**

Intravenous immunoglobulin (IVIg) is currently in clinical study for Alzheimer’s disease (AD). However, preclinical investigations are required to better understand AD-relevant outcomes of IVIg treatment and develop replacement therapies in case of unsustainable supply.

**Methods:**

We investigated the effects of IVIg in the 3xTg-AD mouse model, which reproduces both Aβ and tau pathologies. Mice were injected twice weekly with 1.5 g/kg IVIg for 1 or 3 months.

**Results:**

IVIg induced a modest but significant improvement in memory in the novel object recognition test and attenuated anxiety-like behavior in 3xTg-AD mice. We observed a correction of immunologic defects present in 3xTg-AD mice (−22% CD4/CD8 blood ratio; −17% IL-5/IL-10 ratio in the cortex) and a modulation of CX3CR1^+^ cell population (−13% in the bone marrow). IVIg treatment led to limited effects on tau pathology but resulted in a 22% reduction of the soluble Aβ42/Aβ40 ratio and a 60% decrease in concentrations of 56 kDa Aβ oligomers (Aβ*56).

**Conclusion:**

The memory-enhancing effect of IVIg reported here suggests that Aβ oligomers, effector T cells and the fractalkine pathway are potential pharmacological targets of IVIg in AD.

## Background

The main neuropathological hallmarks of Alzheimer’s disease (AD) are synaptic dysfunction, neuron loss, amyloid plaques, composed of aggregated Aβ peptide, and neurofibrillary tangles, containing hyperphosphorylated forms of the protein tau [1]. Preclinical findings in animal models indicate that active and passive immunization against classical molecular markers of AD might represent suitable therapeutic strategies [2,3]. However, in clinical trials, Aβ-targeted immunotherapies have shown limited efficacy against AD cognitive symptoms [4,5], and have been associated with various adverse effects such as microhemorrhages, vasogenic oedema and aseptic meningitis [6].

Intravenous immunoglobulin (IVIg), which is composed of over 98% human immunoglobulin G (hIgG), is used in the treatment of an increasing number of diseases and is generally safe and well tolerated [7]. Natural autoantibodies against Aβ peptide and oligomers have been reported in the blood of healthy individuals and in IVIg preparations [8,9]. Initial evidence of IVIg efficacy comes from pilot studies in which IVIg improved cognition and reduced Aβ in the cerebrospinal fluid (CSF) in AD patients [10,11]. Results from a large phase III clinical trial (ClinicalTrials.gov identifier: NCT00818662) presented at the 2013 Alzheimer’s Association International Conference indicate that an 18-month treatment with IVIg acted on plasma and positron emission tomography (PET) biomarkers, but did not improve cognitive scores in mild to moderate AD patients at the doses studied [12]. However, subgroup analyses unveiled improved cognitive endpoints in APOE4 carriers [12], suggesting clinical benefit in a subpopulation representing almost 40% of AD patients [13]. These large clinical trials also confirmed the good safety profile of IVIg in AD patients [12,14]. Finally, a retrospective case-controlled study using anonymous medical data indicates that IVIg-treated patients have a 42% lower incidence rate of dementia than an untreated population [15]. Overall, these preliminary data strongly suggest that further clinical studies better powered for subgroup analyses and using larger doses of IVIg are warranted in AD.

Regardless of the results of the clinical assays, the amount that would be needed to treat the 24 million patients afflicted worldwide [16] precludes a widespread use of this product which has to be purified from the plasma of healthy donors. Preclinical research in animal models is thus essential to identify potential pharmacological targets, and to develop a replacement therapy to avoid a massive shortage of plasma that would ensue the use of IVIg as a first-line treatment for AD. The present investigation aimed at replicating the beneficial effects of IVIg in the 3xTg-AD mouse model to decipher its potential mechanisms of action.

## Methods

### Animals and IVIg treatment

Triple-transgenic (3xTg-AD) and age-matching non-transgenic (NonTg) mice were developed by Oddo and colleagues [17] and bred in our animal facility. The 3xTg-AD mouse harbors three mutant genes, namely genes coding for the human beta-amyloid precursor protein (APP_swe_), presenilin-1 (PS1_M146V_) and tau (P301L) [17], and is used as a model for AD since it replicates Aβ and tau pathologies, and displays cognitive deficits. The human APP and tau-independent transgene constructs have been co-injected in embryos harvested from mutant homozygous PS1_M146V_ knock-in mice. Both human APP and tau transgenes are under the control of the Thy1.2 regulatory elements, co-integrated in the same locus and therefore inherited together [17]. The Laval University Animal Research Committee (Québec, QC, Canada) approved all procedures. The treatment and analysis schedule is presented in detail in Table 1. Briefly, 3xTg-AD mice received intraperitoneal administrations of 1.5 g/kg IVIg (Gamunex™; Grifols Canada Ltd., Mississauga, ON, Canada) or the equivalent volume of vehicle (0.2 M glycine pH 4.25, endotoxin free) twice a week for 1 month (nine injections, only for 16-month-old animals at sacrifice) and 3 months (27 injections). Mice were killed at the age of 12 and 16 months. Unless otherwise specified, the age used in the text and Figures refers to the age of the animals at sacrifice at the end of treatment.

**Table 1:** Experimental design

**Age at beginning**	**Treatment duration**	**Group/treatment**	**Age at sacrifice**	**Behavioral tests**	**Postmortem analyses**
**Experiment I**					
9.0 ± 0.1 months of age	3-month treatment	27 i.p. injections (two/week)	12.0 ± 0.1 months of age	Novel object recognition (NOR) task	Brain: ELISA quantification, Western blots, immunohistochemistry and immunofluorescence
		NonTg: ctrl (n *=* 8, 50% F)		Dark-light box emergence test	Blood, whole blood and plasma: saphenous vein blood (after 25 injections), flow cytometry and ELISA
		3xTg-AD: ctrl (n *=* 13, 54% F)/1.5 g/kg IVIg (n *=* 12, 50% F)			Bone marrow, cells: flow cytometry
		Ctrl/vehicle: endotoxin free glycine 0.2 M pH 4.25			Splenocytes: flow cytometry and ELISPOT
**Experiment II**					
12.9 ± 0.2 and 14.8 ± 0.2 months of age	3-month treatment	27 i.p. injections (two/week) (12.9 ± 0.2 months of age)	15.9 ± 0.2 months of age	NOR task	Brain: ELISA quantification, Western blots
		NonTg: ctrl (n *=* 8, 50% F)/1.5 g/kg IVIg (n *=* 8, 50% F)		Barnes maze (3xTg-AD mice, 3-month treatment only)	Blood, whole blood and plasma: intracardiac blood (at sacrifice), flow cytometry and ELISA
		3xTg-AD: ctrl (n *=* 14, 50% F)/1.5 g/kg IVIg (n *=* 13, 46% F)		Open field	
	1-month treatment	Nine i.p. injections (two/week) (14.8 ± 0.2 months of age)			
		NonTg: ctrl (n = 8, 50% F)			
			3xTg-AD: ctrl (n = 14, 50% F)/1.5 g/kg IVIg (n = 13, 46% F)			

IVIg, Gamunex™ (Grifols Canada Ltd., Mississauga, ON, Canada). 3xTg-AD, triple-transgenic mouse model of Alzheimer’s disease; ctrl, control; ELISA, enzyme-linked immunosorbent assay; ELISPOT, enzyme-linked immunosorbent spot; F, female; i.p., intraperitoneal; IVIg, intravenous immunoglobulin; NonTg, non-transgenic; NOR, novel object recognition.

#### Behavioral tests

The effects of IVIg treatment on memory and anxiety-like behavior in 3xTg-AD and NonTg mice were evaluated using a series of cognitive tests. Behavioral tests were performed during the 2 weeks preceding sacrifice with a recovery time of at least 48 hours between every task and a 24-hour delay after the last IVIg injection to reduce stress. The protocols used for all behavioral testing were based on pilot studies previously performed in our colony of 3xTg-AD mice [18,19].

The novel object recognition (NOR) test has been developed to study learning and memory in rodents and is based on their spontaneous tendency to have more interactions with a novel than a familiar object [18]. During the familiarization period, the mouse was placed in a standard cage (29.2 cm × 19 cm × 12.7 cm) containing two identical objects for 5 minutes and returned quickly to its housing cage. Recognition memory was tested 1 hour later by exposing the animal to one familiar and one novel object. The time spent exploring and sniffing each object was recorded. The NOR index was determined as the time spent interacting with the novel object divided by the total time of exploration during the testing phase. Animals whose exploration time was considered insufficient to allow recognition (<10 seconds per object) during the familiarization phase were excluded from analysis.

The dark-light box emergence test was used to evaluate the anxiety-like behavior and was performed as previously described [20]. Mice were initially placed in the center of the dark chamber and had free access to the illuminated chamber. The total time spent in the illuminated chamber and the number of alternation between sides were recorded for 5 minutes. A reduction in the number of alternations or in the time spent in the illuminated compartment was interpreted as increased anxiety.

Mice were tested for spatial memory using a Barnes maze (San Diego Instruments, San Diego, CA, USA) [21]. The 3xTg-AD female mice were tested individually over a 5-day period. Each animal was placed in the center of the maze and subjected to aversive stimuli (bright light and noise). The mouse was given the opportunity to leave the maze through the escape hole. On training days 1 to 4, mice were subjected to 4 × 3-minute trials per day (inter-trial interval time of 20 minutes). For probe trial on Day 5, mice were tested during a 90-second period. Animals were evaluated for their ability to remember the fixed position of an escape compartment. The latency and number of errors before reaching the target hole were recorded for the training and probe phases. For more consistency between animals, all training sessions took place between 7:00 and 12:00 a.m. The mice were subjected to the Barnes maze at the end of the treatment period and the animals were injected with IVIg on Day 2 and 4 of the training session, late in the afternoon to reduce the stress, and sacrificed on Day 5, after the probe trial.

The open field testing measured the general locomotor activity. The open field apparatus consists of ten Plexiglas cages with white translucent walls (80 cm × 80 cm). Movements were tracked by the automated recording of photobeam breaks (San Diego Instruments) to measure horizontal (for example, distance traveled) and vertical activity (for example, rearing). Mice were placed individually in the center of the open field and movements were recorded for 1 hour.

### Reagents and antibodies

All biochemical reagents were purchased from JT Baker (Phillipsburg, NJ, USA) unless otherwise specified. Antibodies used for Western blot and flow cytometry analyses are described in Additional file 1: Table S1.

### Tissue preparation for postmortem analyses

Terminal intracardiac perfusion was performed under deep anesthesia (100 mg/kg ketamine and 10 mg/kg xylazine). For biochemical analyses, parietotemporal cortices were homogenized and centrifuged sequentially to generate a cytosolic fraction (Tris-buffered saline (TBS)-soluble, with protease and phosphatase inhibitors, containing both intracellular and extracellular proteins), a membrane fraction (lysis buffer-soluble (150 mmol/L NaCl, 10 mmol/L NaH_2_PO_4_, 0.5% sodium deoxycholate, 0.5% sodium dodecyl sulfate, 1% Triton X-100), with protease and phosphatase inhibitors, containing both nuclear and membrane-bound proteins) as well as an insoluble fraction (formic acid extract), as previously described [22]. Fractions were kept at −80°C until needed. A commercial enzyme-linked immunosorbent assay (ELISA) was used to quantify Aβ40 and Aβ42 peptides according to the manufacturer (Wako, Osaka, Japan). Protein concentration was determined using the bicinchoninic acid assay (Pierce, Rockford, IL, USA).

Brain cytokine and chemokine concentrations were determined with a multiplex ELISA (Q-Plex™ Mouse Cytokine – Screen (16-plex); Quansys Biosciences, Logan, UT, USA) in parietotemporal cortex homogenates from 12-month-old mice after a 3-month treatment with IVIg and normalized for IL-10 concentration. The following cytokines and chemokines were analyzed: IL-1α, IL-1β, IL-2, IL-3, IL-4, IL-5, IL-6, IL-10, IL-12p70, IL-17, TNFα, granulocyte-macrophage colony-stimulating factor (GM-CSF), regulated on activated, normal T cell expressed and secreted (RANTES), monocyte chemoattractant protein-1 (MCP-1, also called CCL2), IFNγ, and macrophage inflammatory protein 1α (MIP-1α).

For immunofluorescence and immunohistochemistry staining, a brain hemisphere was recovered from female 12-month-old mice, fixed in 4% paraformaldehyde (PFA) for 48 hours and incubated in 20% sucrose at 4°C for >72 hours. The brain was cut on a frozen microtome in 25-μm thick sections and stained. For quantification of amyloid plaques, free-floating sections were first incubated for 15 minutes in 2% H_2_O_2_ and blocked with 1% normal goat serum and 0.4% Triton X-100 in PBS. Sections were then incubated overnight at 4°C, with mouse anti-human APP antibody (clone 6E10). After the overnight incubation, sections were washed in PBS and incubated for 1 hour with biotin-conjugated anti-mouse antibody at room temperature. The sections were further washed and placed in a solution containing an avidin/horseradish peroxidase (HRP) complex (ABC Elite Kit; Vector Laboratories, Burlington, ON, Canada) for 30 minutes at room temperature. The bound peroxidase was revealed with 0.3 mg/mL 3-amino-9-ethylcarbazole (Sigma-Aldrich, St Louis, MO, USA) and 0.03% hydrogen peroxide in acetate buffer. Extensive washings in PBS stopped the reaction. The sections were counterstained with hematoxylin solution, Gill No. 2 (Sigma-Aldrich), and coverslipped with Mowiol mounting media. The area occupied by extracellular staining was quantified in a blinded fashion on five sections of a 1/10 series from bregma, approximately −3.1 mm to −4.1 mm in the mouse brain atlas [23]. At this age, amyloid plaques are present in the hippocampus, mostly in the subiculum. After delineating the subiculum at low magnification (4× objective) the contour of the plaques was performed at higher magnification (10× objective) and the areas were measured using Neurolucida software (MBF Bioscience, Williston, VT, USA). For tau, microglia (ionized calcium-binding adaptor molecule 1 (Iba1)) and APP detection with immunofluorescence labeling, mouse anti-human tau (clone HT7; ThermoFisher Scientific Inc., Pierce Antibodies, Rockford, IL, USA), rabbit anti-Iba1 (Wako) or mouse 6E10 antibodies, respectively, were used as primary antibodies, followed by detection with the appropriate Alexa Fluor-labeled secondary donkey antibody (all from Life Technologies, Burlington, ON, Canada). The nuclei were counterstained with 4′, 6-diamidino-2-phenylindole (DAPI; ThermoFisher Scientific Inc.). Finally, sections were mounted on ColorFrost Plus slides, treated with 0.5% Sudan black (in 70% methanol) for 5 minutes and coverslipped with Mowiol mounting media.

### Flow cytometry

Fluorochrome-conjugated antibodies were used to study the expression of cell surface markers and human IgG in blood, spleen and bone marrow cells (Additional file 1: Table S1). Cells were acquired and analyzed using a CyFlow ML (Partec North America, Inc., Swedesboro, NJ, USA) cytometer and FCS Express software (De Novo Software, Los Angeles, CA, USA).

### ELISPOT

To determine whether the injection of IVIg triggered an anti-human IgG immune response, an enzyme-linked immunosorbent spot (ELISPOT) test was performed, as previously described [24]. Briefly, splenocytes isolated by dissociation of the spleen at sacrifice were unfrozen, washed, counted, plated on human IgG-coated wells (Multiscreen®_HTS_ filter plate; Millipore Corporation, Billerica, MA, USA) and left immobile for antibody secretion. After washing the cells, anti-human-specific mouse immunoglobulins were detected. Each spot was counted under a dissection microscope and considered as a single anti-human IgG-specific B cell clone.

### Statistical analyses

Results for experimental groups are presented as the mean ± SEM. Homogeneity of variances was assessed for all data using the Bartlett’s test. In cases of equal variance, statistical differences were determined using one-way analysis of variance (ANOVA) followed by post-hoc (Tukey’s or Bonferroni’s) tests for comparisons between groups. When homogeneity of variances was rejected, a Welch’s ANOVA followed with the Dunnett’s multiple comparison test was performed. When a Gaussian distribution could not be assumed, non-parametric Kruskal–Wallis or Wilcoxon signed-rank tests were used followed by Dunn’s or Wilcoxon’s post-hoc tests. For comparisons between two groups, a Student’s *t*-test with (for non-homogeneous variances) or without (for homogeneous variances) the Welch’s correction was performed. One sample *t*-test was used to compare the mean NOR index with random chance (that is, 50%). Finally, coefficients of correlation and significance of the degree of linear relationship between two parameters were determined using a simple regression model. The threshold for statistical significance was set to *P <*0.05. All statistical analyses were performed using the JMP (version 9.0.2; SAS Institute Inc., Cary, IL, USA) and Prism 5.0d (GraphPad Software Inc., La Jolla, CA, USA) software.

## Results

### Systemically delivered IVIg distributes to the brain and periphery without inducing a significant immune response

**Figure 1:**
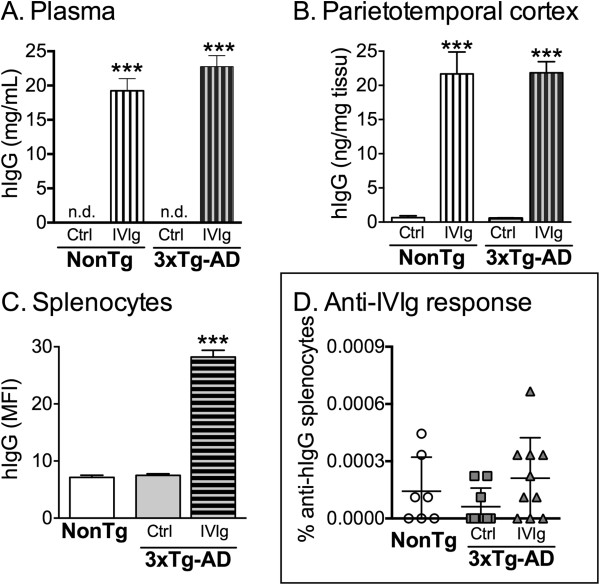
**Biodistribution of IVIg after systemic injections: absence of specific immune response.** Concentrations of human IgG were evaluated in the **(A)** plasma, **(B)** parietotemporal cortex and **(C)** splenocytes of NonTg and 3xTgAD mice after a 3-month treatment with IVIg (1.5 g/kg) or vehicle. The concentrations of IVIg were measured in plasma and parietotemporal homogenates from intracardially-perfused 16-month-old mice, using a specific ELISA. MFI quantification of extracellular human IgG on CD45^+^ splenocytes was used to determine the presence of IVIg in splenocytes of 12-month-old mice. Note that the MFI of the control groups was equivalent to the isotypic control and autofluorescence. Data are presented as the mean ± SEM of 7 to 8 NonTg and 11 to 13 3xTg-AD animals. One-way ANOVA followed by Tukey’s multiple comparison test. ***P <0.001 versus control groups. **(D)** Repeated injections of IVIg did not induce a significant anti-human-specific immune response as shown with human-specific ELISPOT results. Data are presented as the percentage of splenocytes secreting human IgG-specific antibody of 7 to 10 animals treated for 3 months and sacrificed at 12 months of age. Statistics: one-way ANOVA. 3xTg-AD, triple-transgenic mouse model of Alzheimer’s disease; ANOVA, analysis of variance; ctrl, control (vehicle); ELISA, enzyme-linked immunosorbent assay; IgG, immunoglobulin G; IVIg, human intravenous immunoglobulin; MFI, mean fluorescence index; n.d., not detected; NonTg, non-transgenic.

To first evaluate the bioavailability of systemically injected IVIg, hIgG concentrations were measured with a specific ELISA in the brain and plasma of 16-month-old 3xTg-AD and NonTg mice after a 3-month treatment (Figure 1A,B). In the plasma, we observed a mean concentration of 22.8 ± 1.6 mg/mL and 19.4 ± 1.8 mg/mL of hIgG in 3xTg-AD and NonTg mice (Figure 1A), whereas 21.9 ± 1.6 ng/mg tissue and 21.7 ± 3.2 ng/mg tissue were detected in the cortex of 3xTg-AD and NonTg animals, respectively (Figure 1B). Extracellular IVIg was also detected in CD45^+^ splenocytes using flow cytometry analyses, with a mean fluorescence index (MFI) of 28.2 ± 1.2 for 12-month-old treated 3xTg-AD mice compared to 7.5 ± 0.3 for untreated controls (Figure 1C). To evaluate the extent of a possible adaptive immune response to hIgG in treated mice, we next performed ELISPOT analyses. There was no significant increase in the frequency of hIgG-specific antibody-secreting cells among splenocytes (Figure 1D) from 12-month-old 3xTg-AD mice following a 3-month IVIg treatment (0.0002% ± 0.0001% versus 0.0001% ± 0.0001% for IVIg-treated and control 3xTg-AD mice, respectively, *P =* 0.36). Therefore, our results suggest that systemically administered IVIg is distributed in the brain and periphery of treated mice without displaying significant immunogenicity.

### IVIg administration induces a modest improvement in memory impairment and anxiety-like behavior

**Figure 2:**
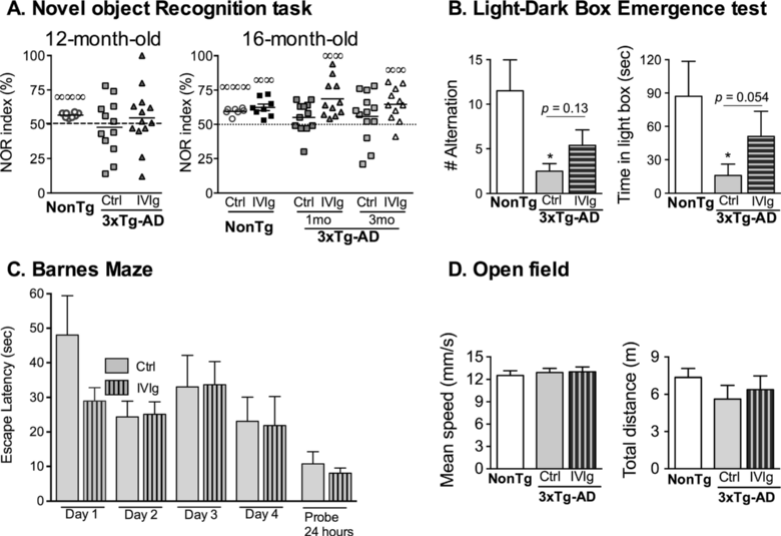
**IVIg treatment improves object recognition memory and anxiety-like behavior in 3xTg-AD mice. (A)** NOR test was performed by 12-month-old (3-month-treatment, n *=* 11 to 12 animals per group) and 16-month-old mice (1- and 3-month-treatments, n *=* 7 to 8 NonTg and n *=* 11 to 13 3xTg-AD animals per group. The mean is indicated with a line. One sample *t*-test. ^∞∞^*P <*0.01, ^∞∞∞^*P <*0.001 versus random chance (50%). **(B)** Dark-light box emergence test was used to evaluate the anxiety-like behavior (n *=* 8 NonTg and n = 11 to 13 3xTg-AD mice per group, 12 months of age after a 3-month treatment; mean ± SEM). Kruskal–Wallis test followed by Wilcoxon’s multiple comparison test, *P <0.05 versus NonTg group. **(C)** Spatial memory deficits were not improved following a 3-month treatment with IVIg as assessed with 16-month-old 3xTg-AD mice exposed to the Barnes maze. Data are presented as mean escape latency ± SEM of 11 to 13 animals per group. Comparisons between groups were performed using a one-way ANOVA followed by Tukey’s multiple comparison test. The area under the curve for the mean escape latency during the training period was also analyzed with a Student’s *t*-test. Furthermore, the mean escape latency during the probe test was compared using a Student’s *t*-test with Welch’s correction. *P* >0.05 in all statistical tests. **(D)** The locomotor performance was evaluated with open field recording in 16-month-old 3xTg-AD mice after a 3-month treatment. The results are shown as the mean ± SEM of n *=* 7 NonTg and n = 11 to 12 3xTg-AD animals per group. 1mo, 1-month treatment; 3mo, 3-month treatment; 3xTg-AD, triple-transgenic mouse model of Alzheimer’s disease; ANOVA, analysis of variance; ctrl, control; IVIg, human intravenous immunoglobulin; NonTg, non-transgenic; NOR, novel object recognition.

To assess the functional consequences of IVIg treatment, 3xTg-AD mice and NonTg littermates were subjected to a battery of behavioral tasks. First, the NOR index was investigated in 12- and 16-month-old 3xTg-AD mice after the administration of IVIg for 1 or 3 months (Figure 2A). IVIg injection significantly ameliorated the NOR index assessed in 16-month-old animals for both treatment durations (NOR index: 64.6 ± 3.7 versus 55.8 ± 4.9 in IVIg and control 3xTg-AD mice treated from 13 to 16 months, respectively) and mitigated the anxiety-like behavior in the dark-light box emergence test in 12-month-old mice (Figure 2B). After a 3-month treatment, the increased anxiety-like behavior usually observed in 3xTg-AD compared to NonTg mice was significant in vehicle-treated, but not in IVIg-treated animals. Indeed, IVIg treatment prolonged the time spent in the illuminated compartment (16.1 ± 10.2 versus 50.9 ± 22.7 seconds for vehicle- and IVIg-treated 3xTg-AD animals, respectively), although the direct comparison between IVIg- and vehicle-treated 3xTg-AD mice did not reach significance threshold (*P =* 0.054). However, the treatment did not improve the performance in the Barnes maze (Figure 2C). Interestingly, whereas the Barnes maze tests the spatial memory, the NOR index reflects the recognition memory, two distinct cognitive functions that were differently affected by IVIg treatment in this study. To rule out a possible correction of motor deficits by IVIg as the main cause of these effects, the locomotor activity of the animals was monitored with an open field test, which showed no difference between the various groups (Figure 2D).

### Effects of IVIg on Aβ, tau and synaptic pathologies

**Figure 3:**
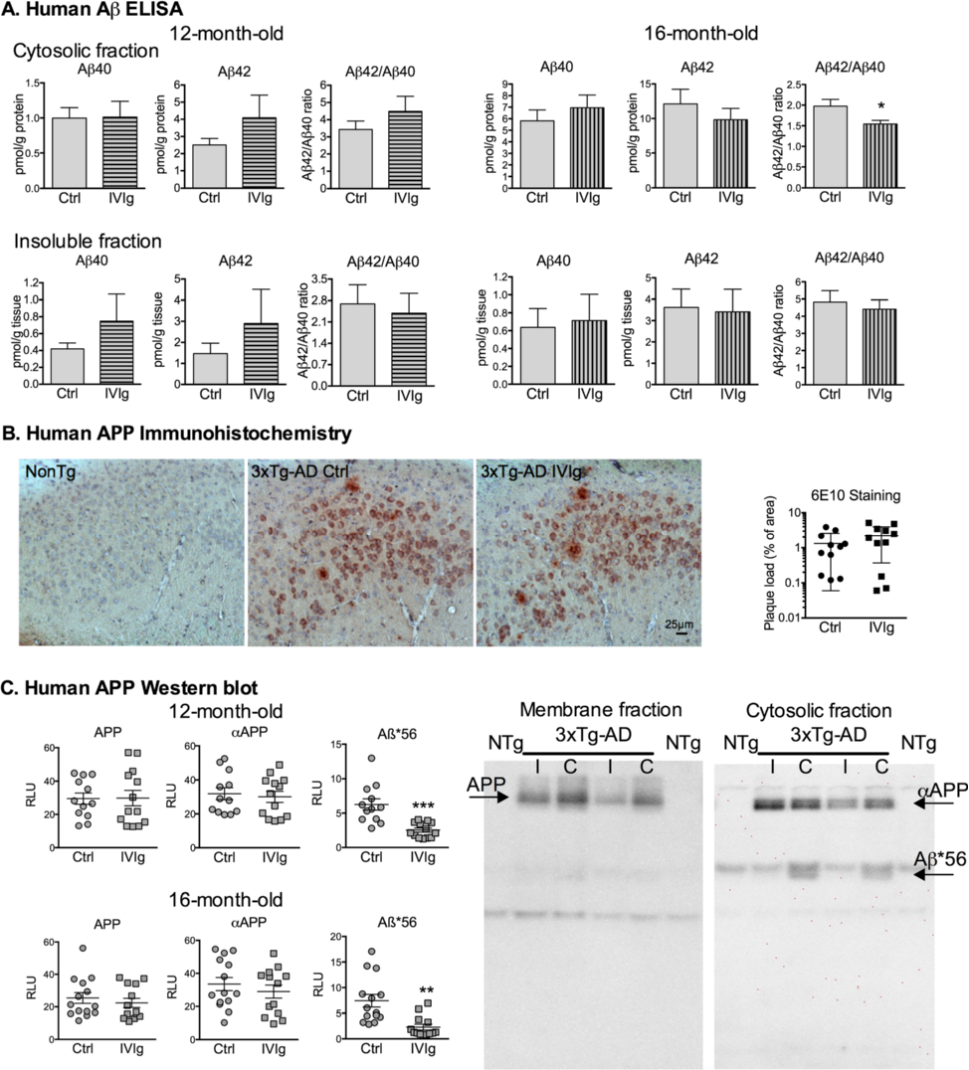
**IVIg treatment decreases Aβ42/Aβ40 ratio and 56 kDa Aβ oligomers. (A)** Human Aβ_40_ and Aβ_42_ peptides were quantified using specific ELISA in parietotemporal cortex homogenates of 3xTg-AD mice. Aβ concentrations in soluble and insoluble fractions remained unchanged following IVIg injections in 12-month-old (3-month treatment, n *=* 11 to 13 mice) and 16-month-old 3xTg-AD mice at sacrifice (pooled 1- and 3-month-treatment duration, n *=* 25 animals). The Aβ42/Aβ40 ratio was significantly decreased in the soluble fraction of 16-month-old IVIg-treated mice. **(B)** Aβ deposition was analyzed in the hippocampus after immunohistochemistry using 6E10 antibody in NonTg and 3xTg-AD mice. 20× magnification. Right: graph shows the quantification of the area occupied by extracellular amyloid plaques in four consecutive brain sections of the subiculum of the hippocampal formation from 12-month-old 3xTg-AD mice treated for 3 months with IVIg or vehicle (n = 11 per group). Red, human APP; blue, hematoxylin counterstain. **(C)** The expression of transgenic human APP (membrane fraction) and the soluble αAPP fragment and Aβ*56 oligomers (cytosolic fraction) were quantified by Western blot analysis with 6E10 antibody in the cortex of 12- and 16-month-old mice treated for 3 months with IVIg (n = 13 animals) or vehicle (n = 12 to 14 animals). Significant reduction of soluble Aβ*56 oligomers was also observed on IVIg treatment. Examples of immunoblots are shown for both fractions (bottom right). Values are expressed as mean ± SEM. Statistical analysis: unpaired Student *t*-test with Welch’s correction. *P <0.05, **P <0.01, ***P <0.001 versus vehicle-treated 3xTg-AD mice. 3xTg-AD, triple-transgenic mouse model of Alzheimer’s disease; APP, amyloid precursor protein; C, control; ctrl, control; ELISA, enzyme-linked immunosorbent assay; I, human intravenous immunoglobulin; IVIg, human intravenous immunoglobulin; NonTg, non-transgenic; NTg, non-transgenic; RLU, relative luminescence unit.

The presence of natural anti-Aβ and anti-tau antibodies in IVIg preparations has been proposed as a potential mechanism by which IVIg could serve as a broad-range, passive immunization agent [8]. As anti-Aβ antibodies successfully reduced Aβ and tau pathologies along with cognitive impairment in several animal models [2], we hypothesized that IVIg might share the same mechanism. Concentrations of soluble and insoluble Aβ40 and Aβ42 peptides were first determined in homogenates of parietotemporal cortex with a specific ELISA. When pooling the results from both the 1- and 3-month treatments, IVIg administration led to a 22% reduction of the soluble Aβ42/Aβ40 ratio in 16-month-old, but not in 12-month-old mice at sacrifice, without any significant effect on insoluble Aβ levels (Figure 3A). In the 16-month-old animals, two-way ANOVA statistical analysis also revealed a significant reduction of the Aβ42/Aβ40 ratio following IVIg treatment (*P* = 0.0293). It is noteworthy that only the groups with the reduced Aβ42/Aβ40 showed an improved NOR index. Consistent with the later absence of effects on insoluble Aβ, quantification of extracellular amyloid deposition with 6E10 immunohistochemistry showed no impact of IVIg on plaque accumulation (Figure 3B). The production of Aβ is the result of the selective cleavage of the membrane protein APP by α-, β- and γ-secretase into soluble αAPP and Aβ peptide. Furthermore, the Aβ peptide is prone to oligomerization into dimers, trimers and dodecamers. Therefore, we also assessed the effect of IVIg on concentrations of total membrane APP, soluble αAPP and Aβ dodecamer (Aβ*56) by immunoblot analysis in homogenates of parietotemporal cortex, using the 6E10 antibody [25]. The levels of APP and αAPP were stable, but a loss of over 60% Aβ*56 was found after a 3-month treatment with IVIg in 3xTg-AD mice at both ages, compared to vehicle-treated animals (Figure 3C).

**Figure 4:**
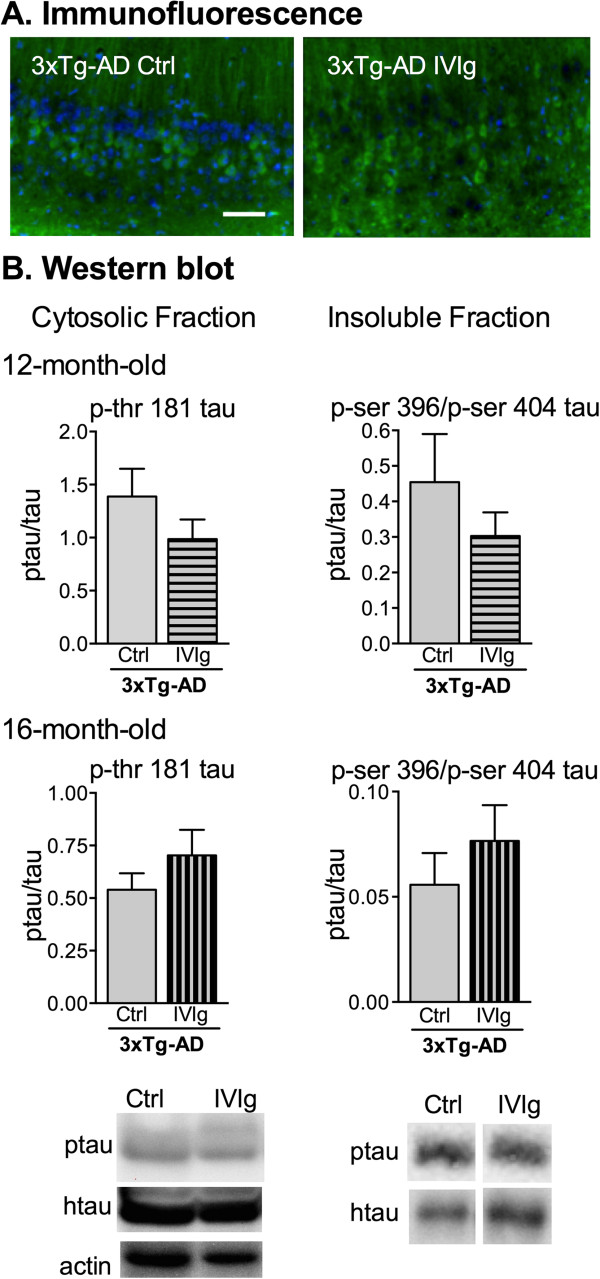
**Lack of effects of IVIg on tau pathology in 3xTg-AD mice. (A)** Brain sections of 12-month-old mice at sacrifice (n *=* 3 per group) were stained for tau using anti-human tau antibody (HT-7) after a 3-month treatment with IVIg or vehicle (ctrl) and imaged with fluorescence. Sections shown are from the CA1 region of the hippocampus (bregma approximately −3.5). Scale bar = 100 μm. Green, human tau; blue, DAPI. **(B)** Immunoblot analyses were performed on protein fractions from the parietotemporal cortex of 3xTg-AD mice. Ratios of phosphorylated tau/human total tau showed the absence of protective effects of IVIg injections. Total human tau and phosphorylated tau were analyzed in the cytosolic (left) and insoluble fractions (right). Representative results are presented for mice treated for 3 months and sacrificed at the age of 12 months and 16 months (n *=* 10 to 13 animals per group). Values are expressed as the mean ± SEM. Statistical analysis: unpaired Student *t*-test with Welch’s correction. Examples of immunoblots in both fractions (bottom). The homogeneity of sample loading in cytosolic fraction was determined by protein quantification and actin staining. 3xTg-AD, triple-transgenic mouse model of Alzheimer’s disease; ctrl, control; DAPI, 4′, 6-diamidino-2-phenylindole; IVIg, human intravenous immunoglobulin.

Since AD is also associated with severe tau pathology and loss of synaptic markers [1], we evaluated the effect of the IVIg treatment on these pathologies. We first immunostained 3xTg-AD brains for phosphorylated tau. Although characteristic immunostaining was observed in the 3xTg-AD (Figure 4A) but not the NonTg brains, we failed to detect significant differences in the intensity of the immunoreactivity. Total tau and several phosphorylated isoforms were quantified in the cytosolic and insoluble fractions of 12- and 16-month-old mice (Figure 4B), without any consistent effect of IVIg. Similarly, synaptic protein concentrations (Table 2), as measured by immunoblots in the cytosolic and membrane of parietotemporal cortices, remained unchanged by IVIg treatments. These data suggest a role for IVIg in preventing the accumulation of toxic Aβ oligomers along with limited activity on non-amyloid pathological hallmarks of AD.

**Table 2:** Levels of proteins in the parietotemporal cortex of 3xTg-AD mice

**Biological function**	**Proteins**	**Age group**	**Fraction**	**NonTg ctrl**	**3xTg-AD ctrl**	**3xTg-AD IVIg**	**ANOVA *P *** **value**
Synaptic	Snap25	12 months	Membrane	1.58 ± 0.38	1.48 ± 0.4	1.46 ± 0.38	0.78
	PSD95	12 months	Membrane	1.31 ± 0.33	1.39 ± 0.31	1.39 ± 0.33	0.85
	Septin3	16 months	Cytosolic	1.16 ± 0.17	1.55 ± 0.38*	1.60 ± 0.42*	**0.04**
	Dynamin1	16 months	Membrane	1.56 ± 0.23	1.38 ± 0.15	1.50 ± 0.12	0.75
	Drebrin	16 months	Membrane	1.35 ± 0.24	1.57 ± 0.55	1.63 ± 0.41	0.36
	Synaptophysin	16 months	Membrane	6.73 ± 1.5	6.86 ± 1.13	6.82 ± 1.16	0.97
Others	VILIP-1	12 months	Cytosolic	3.01 ± 0.59	2.68 ± 0.42	2.58 ± 0.35	0.10
	VILIP-1	16 months	Cytosolic	1.74 ± 0.19	1.58 ± 0.31	1.58 ± 0.25*	**0.01**
	ADAM-10	12 months	Cytosolic	8.08 ± 0.45	7.38 ± 0.23	7.55 ± 0.39	0.43
	ADAM-10	16 months	Cytosolic	7.97 ± 0.51	8.27 ± 0.37	7.36 ± 0.24	0.27
	PAK	16 months	Cytosolic	8.05 ± 2.49	6.81 ± 0.71	7.50 ± 0.93	0.07

Western blot analyses. Equal loading of samples was determined by protein quantification and actin staining. Values are expressed as the mean ± SEM of RLU (n = 7 to 13 animals). Statistical analysis: one-way ANOVA followed with Tukey’s multiple comparison test. *P <0.05 versus NonTg ctrl; ^§^*P <*0.05 versus 3xTg-AD ctrl. 3xTg-AD, triple-transgenic mouse model of Alzheimer’s disease; ADAM-10, a disintegrin and metalloproteinase domain containing protein 10; ANOVA, analysis of variance; ctrl, control; IVIg, human intravenous immunoglobulin; NonTg, non-transgenic; PAK, p21-activated kinase; PSD95, postsynaptic density protein 95; RLU, relative luminescence unit; Snap25, synaptosomal-associated protein 25; VILIP-1, visinin-like protein-1.

### IVIg corrects the immunological defects in 3xTg-AD mice and decreases CX3CR1 cell populations in the bone marrow

**Figure 5:**
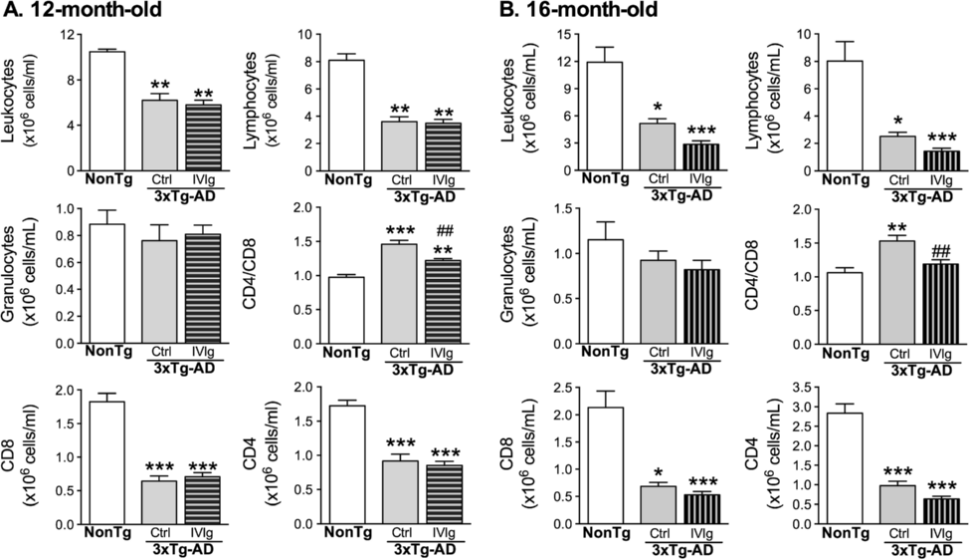
**IVIg injection modulates the relative populations of T lymphocytes.** Flow cytometry analysis of blood cells labeled for leukocytes (CD45), lymphocytes (B220 for B cells and CD3 for T cells), helper (CD4) and cytotoxic (CD8) T cells, and granulocytes (Gr-1). Mice were exposed to IVIg for 3 months and sacrificed at the age of **(A)** 12 months and **(B)** 16 months. Results are presented as the mean ± SEM (n = 8 NonTg and n = 11 to 13 3xTg-AD animals per group). Statistical analysis: one-way ANOVA followed by Tukey’s multiple comparison test. *P <0.05, **P <0.01, ***P <0.001 versus NonTg group; ^##^*P <*0.01 versus control 3xTg-AD. 3xTg-AD, triple-transgenic mouse model of Alzheimer’s disease; ANOVA, analysis of variance; ctrl, control; IVIg, human intravenous immunoglobulin; NonTg, non-transgenic.

**Figure 6:**
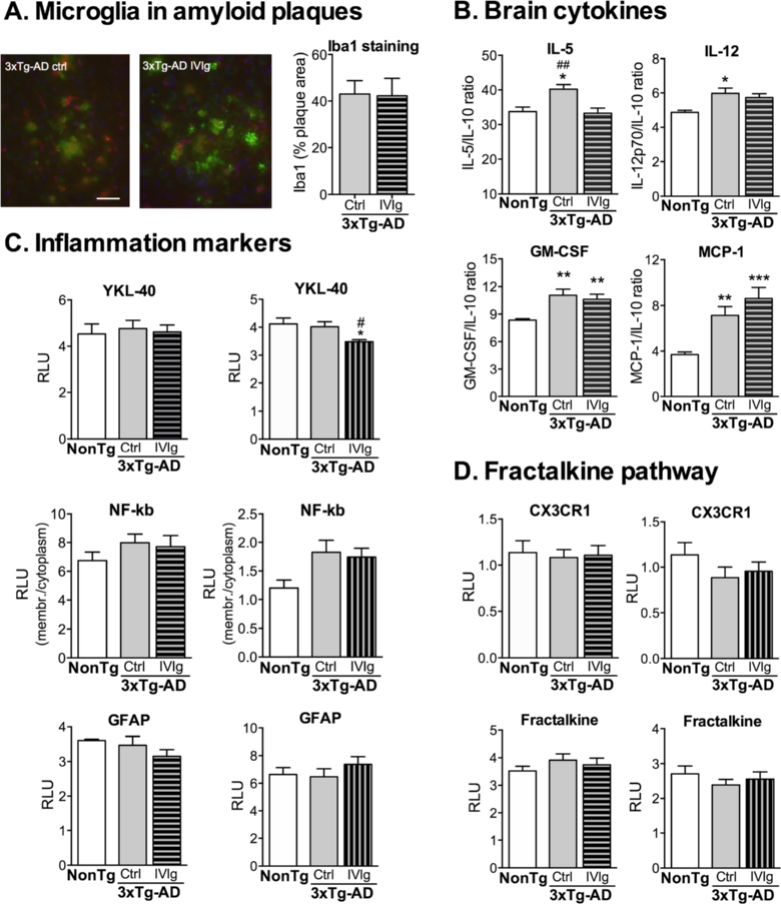
**Neuroimmunomodulation following IVIg treatment. (A)** Brain sections of 12-month-old female 3xTg-AD mice (3-month treatment with IVIg or ctrl) were stained and imaged with fluorescence. Iba1 (microglial cells, red), human APP/Aβ (6E10 antibody, green), DAPI (blue). Sections shown are from the subiculum of the hippocampal formation. Blue, DAPI. Scale bar = 35 μm. Right: graph of the area occupied by Iba1 staining in amyloid plaques (n = 4 per group). Student *t*-test. *P =* 0.94. **(B)** Multiplex ELISA was used to quantify the cytokine levels in the parietotemporal cortex of 12-month-old mice (3-month treatment). The values were normalized to IL-10 concentrations (n *=* 7 to 8 NonTg, n = 10 to 12 ctrl and n = 12 to 13 IVIg animals per group). Data were analyzed using a Kruskal–Wallis test followed by Dunn’s multiple comparison test. *P <0.05, **P <0.01, ***P <0.001 versus NonTg group; ^##^*P <*0.01 versus 3xTg-AD control group. **(C)** Inflammation markers and **(D)** fractalkine pathway in the cortex of mice treated with IVIg. One-way ANOVA with Tukey’s multiple comparison test. *P <0.05 versus NonTg group; ^#^*P <*0.05 versus 3xTg-AD control group. (C,D) Immunoblot analyses. Left panels: mice treated from 9 to 12 months (n = 8 NonTg, n = 12 ctrl and n = 13 IVIg animals per group). Right panels: mice treated from 13 to 16 months (n = 7 to 8 NonTg, n = 12 to 13 ctrl and n = 13 to 14 IVIg animals per group). 3xTg-AD, triple-transgenic mouse model of Alzheimer’s disease; APP, amyloid precursor protein; ctrl, control (vehicle); DAPI, 4′, 6-diamidino-2-phenylindole; ELISA, enzyme-linked immunosorbent assay; GFAP, glial fibrillary acidic protein; GM-CSF, granulocyte-macrophage colony-stimulating factor; Iba1, ionized calcium-binding adaptor molecule 1; IL, interleukin; IVIg, human intravenous immunoglobulin; MCP-1, monocyte chemoattractant protein-1; NF-κb, nuclear factor-kappa B; NonTg, non-transgenic.

A growing number of studies show an association between neurodegenerative processes and immune changes, both in the central nervous system (CNS) and in the periphery [26], and genetic studies indicate that markers of immunity such as variants of complement receptor 1, triggering receptor expressed on myeloid cells 2 (TREM2) and clusterin genes are risk factors for late-onset AD [27-29]. More specifically, systemic immunity and inflammation are involved in progression of the pathology in the 3xTg-AD model [30]. Therefore, we investigated the peripheral leukocyte cell populations in the blood of 3xTg-AD mice. Compared with NonTg mice, total leukocytes and lymphocytes were decreased in this model at 12 and 16 months of age (Figure 5A,B). However, administration of IVIg for 3 months corrected the increase of the CD4/CD8 T cell ratio observed in the blood of 12- and 16-month-old 3xTg-AD versus NonTg mice (Figure 5). We next analyzed markers of brain immune response in IVIg-treated 3xTg-AD animals. We first observed microglial activation in the vicinity of amyloid plaques in both control and IVIg-treated 3xTg-AD mice (Figure 6A), as previously reported in the same model [31,32]. Multiplex ELISA probing unveiled higher levels of pro-inflammatory cytokines IL-5, IL-12, GM-CSF and MCP-1 in the cortex of 12-month-old 3xTg-AD mice, compared to NonTg, when expressed as ratios relative to the anti-inflammatory cytokine IL-10 (Figure 6B). Interestingly, following a 3-month IVIg treatment, IL-5/IL-10 and IL-12/IL-10 cortical ratios in 3xTg-AD mice were brought back to levels comparable to those in NonTg animals (Figure 6B). Although markers of astrogliosis (glial fibrillary acidic protein (GFAP)) and the transcription factor nuclear factor-kappa B (NF-κb) remained unaffected by the IVIg treatment, in 16-month-old mice, it significantly decreased the levels of chitinase 3-like protein 1 (YKL-40), a protein possibly involved in the neuroinflammatory response [33], as determined by immunoblot analyses of cortex extracts from 3xTg-AD mice (Figure 6C). Taken together, these data thus point to a positive action of IVIg in the control of immune homeostasis.

**Figure 7:**
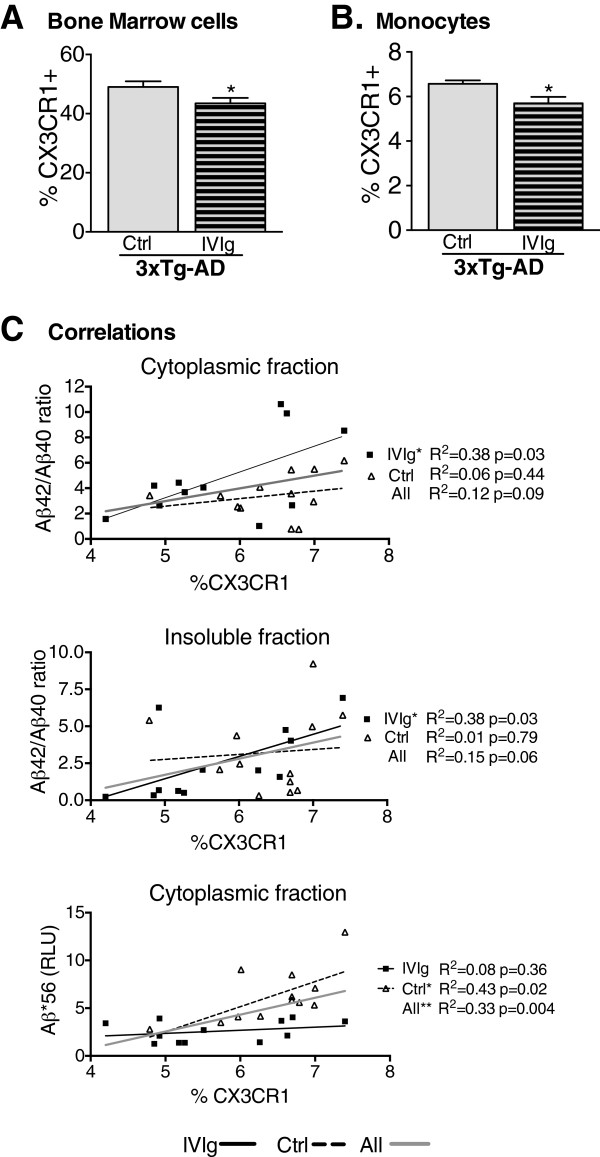
Modulation of the fractalkine pathway by IVIg treatment: correlation with cortical Aβ42/Aβ40 ratios and Aβ*56. Expression of CX3CR1 was evaluated using flow cytometry in the bone marrow of 3xTg-AD mice treated with IVIg from 9 to 12 months. Decreases in the percentage of CX3CR1^+^ cells in **(A)** total bone marrow cells and **(B)** the monocyte population were observed in treated animals (n *=* 11 to 12). **(C)** The percentage of CX3CR1^+^ cells in the bone marrow correlated with soluble and insoluble Aβ42/Aβ40 ratios (in IVIg-treated mice) and Aβ*56 concentrations from the parietotemporal cortex (in control and total animals). Statistical analysis: (A,B) one-way ANOVA with Tukey’s multiple comparison test. *P <0.05. (C) Correlations were determined by simple linear regression analysis. 3xTg-AD, triple-transgenic mouse model of Alzheimer’s disease; ANOVA, analysis of variance; IVIg, human intravenous immunoglobulin.

The fractalkine receptor CX3CR1 is expressed in specific subsets of lymphocytes, natural killer cells and monocytes [34]. The impact of CX3CR1 on neurodegeneration is still debated, but previously published data demonstrate a reduction of amyloid deposits and neuronal loss, and increased *in vitro* phagocytosis in knockout animals for CX3CR1 [35-37]. When measured by Western blot analysis, expression levels of CX3CR1 and its ligand, fractalkine, were not modulated in the cortex of 3xTg-AD mice following a 3-month treatment with IVIg (Figure 6D). However, flow cytometry analyses revealed a 13% decrease in total CX3CR1^+^ cells in the bone marrow from 3xTg-AD mice treated from 9 to 12 months of age (Figure 7A). Consistent with this, an 11% decrease in the percentage of CX3CR1^+^ monocytes was also observed following the same treatment (Figure 7B). Intriguingly, this reduction was correlated with changes in soluble and insoluble Aβ42/Aβ40 ratios as well as Aβ*56 concentration in the brain (Figure 7C), implying that bone marrow cells with the decreasing expression of CX3CR1 might be linked to the reduction of cortical Aβ pathology. Such a modulation of fractalkine signaling may represent a pathway through which IVIg exerts its effects and support a pharmacological intervention targeting CX3CR1 in AD.

## Discussion

Our results are consistent with IVIg-induced improvement of behavioral function, reduction of Aβ*56 oligomer levels and immunomodulation in the 3xTg-AD mouse model, without altering the non-amyloid aspects of AD neuropathology. To our knowledge, this is the first demonstration that chronic administration of IVIg can strikingly decrease levels of the pathogenic oligomer Aβ*56 in association with reduced expression of peripheral CX3CR1 and attenuation of behavioral deficits in a mouse model of AD. IVIg also displayed strong immunomodulatory properties, leading to a correction of immune abnormalities frequently observed in AD and animal models.

The use of IVIg in AD was initially motivated by the hypothesis that it contains natural, polyclonal, conformation-specific antibodies against Aβ. This view is supported by the lower titer of anti-Aβ antibodies found in the blood of AD patients compared to controls [8,9]. We thus analyzed the impact of IVIg on various parameters of brain amyloid pathology and found no significant reduction of either Aβ40 or Aβ42 in both soluble and insoluble protein fractions from treated mice, consistent with a recent report in which IVIg treatment in the AβPPswe/PS1ΔE9 mouse model of AD failed to decrease Aβ concentrations in the hippocampus [38]. However, we observed a 22% decrease in the soluble Aβ42/Aβ40 ratio following IVIg treatment in 16-month-old 3xTg-AD mice. This finding is interesting in view of the fact that, in familial AD, most known APP mutations increase the Aβ42/Aβ40 ratio without necessarily changing the total concentration of Aβ peptides formed, shifting the proteolysis of APP in favor of Aβ42, which is more prone to oligomerization [39]. Furthermore, an *in vitro* study of APP and Aβ processing in familial AD indicates that the Aβ42/Aβ40 ratios correlate inversely with the age of onset of AD [40]. In the Tg2576 mouse, a reduction of spine density, a decline in long-term potentiation, fear conditioning impairments and an increase in Aβ42/Aβ40 ratio precede amyloid plaque deposition [41]. Moreover, an approximate 30% increase in the insoluble Aβ42/Aβ40 ratio is associated with spatial memory deficits following a partial loss of glutamate transporter 1 in the AβPPswe/PS1ΔE9 mouse model [42]. Consistent with these findings, a substantial decrease in the soluble Aβ*56 oligomer species was also observed in IVIg-treated 3xTg-AD mice. There is no consensus on the actual relevance and toxicity of the various Aβ oligomers associated with AD pathogenesis. The Aβ*56 species are found at the AD synapses [43] and are elevated in the CSF of cognitively normal adults at greater risk for AD [44]. In animal models, intracerebral administration of Aβ*56 produces cognitive impairments in a concentration-dependent manner [45,46]. In addition, Aβ*56 levels show a better association with learning/memory deficits than plaque load [25] in most transgenic AD models. Finally, in cognitively intact elderly subjects, Aβ*56 correlates positively with soluble pathological tau species and negatively with the postsynaptic proteins, drebrin and fyn kinase, suggesting that Aβ*56 may play a pathogenic role very early in the pathogenesis of AD [47]. The present data, in line with lower incidence rate of dementia in IVIg-treated patients [15], suggests that IVIg impedes accumulation of Aβ oligomers possibly by an effect on their production, aggregation, degradation or clearance, and might prevent AD in the pre-clinical stage. Furthermore, although not significant in our study, Puli and colleagues [38] reported a significant rise in the soluble levels of Aβ40 and Aβ42 peptides in the AβPPswe/PS1ΔE9 mouse model following an 8-month treatment with IVIg that would be consistent with decreased Aβ oligomer/monomer ratio following IVIg injections.

In addition to its anti-Aβ action, it can be hypothesized that the immunomodulatory effect of IVIg contributes to its effect in the CNS [8]. Indeed, IVIg administration increases C5a brain levels [48] and reduces the expression of the CD45 marker in a sub-population of microglial cells in mice, in association with increased neurogenesis [38]. We found that chronic IVIg treatment steadily decreases the CD4/CD8 cell ratio in 3xTg-AD mice, as previously reported in a mouse model of Parkinson’s disease [24]. Such a decrease in the CD4/CD8 cell ratio was also reported in IVIg-treated patients [49], suggesting that it may actually provide a clinically relevant index of IVIg efficacy. Interestingly, the natalizumab-induced decrease in CD4/CD8 ratio in multiple sclerosis patients is significantly related to clinical response [50,51]. In AD patients, however, the actual relevance of the CD4/CD8 ratio is still much debated. Indeed, increased [52,53], unchanged [54] and even decreased [55] CD4/CD8 ratios have been reported in AD patients compared to controls. Thus, the clinical implications for AD progression of the IVIg-induced decrease of the CD4/CD8 ratio observed here remain to be further established. We also report evidence that IVIg decreases the protein levels of YKL-40 in the cortex of older mice. Although the function of YKL-40 is still under study, it has been associated with local neuroinflammation in acute and chronic diseases [33], and is increased in the CSF of AD patients [56]. We also report altered pro/anti-inflammatory ratios of cytokines and chemokines in 3xTg-AD mice. In agreement with the extensive body of evidence showing an imbalance in cytokine and/or chemokine production in the blood, CSF or brain of individuals diagnosed with AD [57], we observed a rise in the ratios of IL-5, IL-12, MCP-1 and GM-CSF over IL-10 in the brain of 3xTg-AD mice. Furthermore, although IVIg treatment had no effect on GM-CSF/IL-10 and MCP-1/IL-10, it reduced both IL-5/IL-10 and IL-12/IL-10 ratios. Atopy is a genetic predisposition to hypersensitivity reactions against common environmental allergens, which manifests itself as asthma, dermatitis or rhinitis [58]. Whereas an increased IL-5/IL-10 ratio has been reported in patients with atopic asthma [59], atopy itself is associated with a modest rise in the risk of dementia [58], in support of an inflammatory component in the etiology of neurodegenerative dementia.

Finally, recent studies have linked the anti-inflammatory effects of IVIg to terminal α2,6-linked sialic acid residues on the N-linked glycans of a sub-population of the IgG fragment crystallizable (Fc) domain [60-62]. These sialylated IgG bind to the human receptor dendritic cell-specific intercellular adhesion molecule-3-grabbing non-integrin (DC-SIGN) or its murine orthologue, specific intracellular adhesion molecule-grabbing non-integrin receptor 1 (SIGN-R1) and, in animal models of autoimmune diseases, this interaction leads to the expression of anti-inflammatory cytokines and receptors [61]. Therefore, sialylated IgG might recapitulate the immunosuppressive action of IVIg. If confirmed, the sialylated fraction could provide a suitable alternative to this blood-derived product. Interestingly, the SIGN-R1 receptor is also expressed on mouse microglia [63] and therefore could interact with sialylated IVIg in the brain. However, it is noteworthy that the results of IVIg treatment in the 3xTg-AD model were not limited to immunosuppression suggesting that sialylated IVIg fractions could hardly account for all the effects observed on AD markers. Taken together, these observations support the view that IVIg acts, at least in part, on maintaining brain immune homeostasis, providing a favorable environment against neurodegenerative diseases.

The interaction between CX3CR1 and its exclusive ligand fractalkine is required for the physiological trafficking of circulating monocytes to organs, and is an important regulator of autoimmune inflammation and antigen-specific leukocyte recruitment from the blood and the bone marrow to the CNS [34]. Interestingly, IVIg is used in the treatment of idiopathic thrombocytopenic purpura and increased expression of CX3CR1 has been observed in these patients [7,64]. Thus, our data support the reduction of CX3CR1 expression on peripheral leucocytes as a new mechanism of action for IVIg. Studies on the role of the fractalkine pathway in animal models of AD have generated somewhat contradictory results. CX3CR1 genetic depletion was found to protect from neuronal loss in the 3xTg-AD model [37] and reduce β-amyloid deposition by activating its phagocytosis in CRND8, APPPS1 and R1.40 mice [35,36], whereas it exacerbates tau phosphorylation and aggregation, and enhances cognitive deficits in hTau and hAPP mice [65,66]. These results strongly suggest that CX3CR1 signaling regulates microglial activity and neuropathological processes in AD. However, genetic knockouts of CX3CR1 hardly mimic the actual trends in CX3CR1 expression in physiological conditions and during the progression of the disease. In the brain, the fractalkine pathway mediates the communication between neurons, which produce fractalkine, and microglia, which express CX3CR1, as binding of the two inhibits microglial activation [67]. In the present study, IVIg administration had no effect on brain expression levels of CX3CR1 or fractalkine, as detected by Western blot analysis. However, our data show an 11 to 13% decrease in CX3CR1^+^ cells in the bone marrow from IVIg-treated 3xTg-AD mice, which was correlated with a reduction of both Aβ*56 concentration and Aβ42/Aβ40 ratios in the cortex. A recent study by Smolders and colleagues [68] reported an enrichment of CD8+ cells in the corpus callosum in humans and associated CD4/CD8 ratio reduction. This increase in CD8+ T cells was associated with higher expression of the CX3CR1 chemokine receptor and the authors suggested that it could serve in the homing of T cells to the brain parenchyma. The fact that the concentration of T cells is low in the brain could explain our failure to detect an increase in CX3CR1 protein levels in the IVIg-treated mice. Therefore, the fractalkine pathway clearly warrants further investigation as a therapeutic target of IVIg or other compounds in neurodegenerative diseases.

## Conclusion

**Figure 8:**
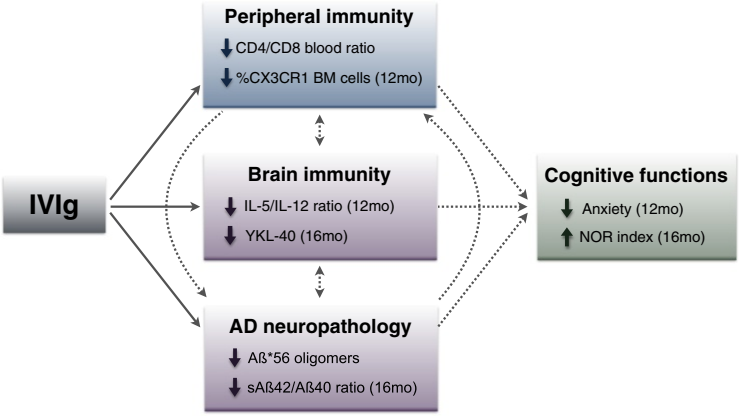
**A model for IVIg mechanisms of action in the 3xTg-AD mice.** Our observations suggest that IVIg can act on cerebral and peripheral immunity, markers of AD neuropathology and cognitive functions. Systemic injections of IVIg modulate the peripheral immunity and activate phagocytes by decreasing CX3CR1^+^ expression. These peripheral immune cells can potentially migrate to the brain where they can restore the immune homeostasis of the CNS. Alternatively, IVIg might directly enter the brain, attenuate neuroinflammation and provide a favorable environment against neurodegenerative diseases. In the CNS, IVIg could also directly impact AD pathology by modulating the metabolism of Aβ (production, aggregation, degradation or clearance). Together, correction of immunologic imbalance and decreased AD pathology could provide a favorable outcome on recognition memory and anxiety. Furthermore, these effects of IVIg in the 3xTg-AD mice support a multi-target action in AD, although further study is needed to dissect the therapeutic value of potential pharmacologic substitutes. Dotted line, speculative links; solid line, results from IVIg treatment. ↓, reduction; ↑, increase. 3xTg-AD, triple-transgenic mouse model of Alzheimer’s disease; AD, Alzheimer’s disease; CD, cluster of differentiation; CNS, central nervous system; CX3CR1, C-X3-C chemokine receptor 1; IL, interleukin; IVIg, intravenous immunoglobulin; mo, months of age; NOR, novel object recognition; sAβ42/40, soluble Aβ42/40; YKL-40, chitinase 3-like protein 1 (CHI3L1).

Taken together, our results show that IVIg treatment ameliorates cognitive performance, decreases Aβ42/Aβ40 ratios and Aβ*56 concentrations in the brain, reduces inflammation, and modulates the expression of CX3CR1 in the bone marrow (Figure 8). These data are consistent with biomarker analyses from the recent phase III trial on IVIg where a dose-dependent decrease in plasmatic Aβ42 and a reduction in PET-determined [18 F]florbetapir binding in the brain were reported [12]. In keeping with the recent failure of anti-Aβ-based immunotherapies to improve cognition in clinical trials [5], regulation of Aβ pathology may not be sufficient to fully account for the action of IVIg in AD, particularly in the absence of an amelioration of tau pathology and synaptic defects. Thus, a combination of these effects on Aβ pathology and immune parameters is likely responsible for the cognitive improvements detected in IVIg-treated 3xTg-AD mice. More specifically, the present data provides important insights into the mechanism of action of IVIg in AD and identifies Aβ*56 oligomers, effector T cells and fractalkine signaling as possible targets for a pharmacological substitute for the severely limited supply of IVIg available for AD therapy.

## Abbreviations

3xTg-AD: Triple-transgenic mouse model of Alzheimer’s disease; Aβ: Amyloid-beta peptide; Aβ*56: 56KDa Aβ oligomer; AD: Alzheimer’s disease; ANOVA: Analysis of variance; APP: Amyloid precursor protein; CD: Cluster of differentiation; CNS: Central nervous system; CSF: Cerebrospinal fluid; ctrl: Control (vehicle); CX3CR1: C-X3-C chemokine receptor 1; CX3CL1: C-X3-C motif ligand 1 (fractalkine); DAPI: 4′, 6-diamidino-2-phenylindole; DC-SIGN: Dendritic cell-specific intercellular adhesion molecule-3-grabbing non-integrin; ELISA: Enzyme-linked immunosorbent assay; ELISPOT: Enzyme-linked immunosorbent spot; Fc: Fragment crystallizable; GFAP: Glial fibrillary acidic protein; GM-CSF: Granulocyte-macrophage colony-stimulating factor; hIgG: Human immunoglobulin G; HRP: Horseradish peroxidase; Iba1: Ionized calcium-binding adaptor molecule 1; IFN: Interferon; IgG: Immunoglobulin G; IL: Interleukin; IVIg: Intravenous immunoglobulin; MCP-1: Monocyte chemoattractant protein-1 (CCL2); MFI: Mean fluorescence index; MIP: Macrophage inflammatory protein; NF-κb: Nuclear factor-kappa B; NonTg: Non-transgenic mouse; NOR: Novel object recognition; PBS: Phosphate-buffered saline; PET: Positron emission tomography; PFA: Paraformaldehyde; PS1: Presenilin-1; RANTES: Regulated on activated normal T cell expressed and secreted; SEM: Standard error of the mean; SIGN-R1: Specific intracellular adhesion molecule-grabbing non-integrin receptor 1; TBS: Tris-buffered saline; TNF: Tumor necrosis factor; TREM2: Triggering receptor expressed on myeloid cells 2; YKL-40: Chitinase 3-like protein 1 (CHI3L1).

## Competing interests

FC and RB have received funding from Grifols (Mississauga, ON, Canada). The funding sources had no involvement in the study design, and in the collection, analysis or interpretation of the data. The remaining authors declare that they have no competing interests.

## Authors’ contributions

ISA designed the experiments, performed the animal studies and most of the postmortem analyses, analyzed the data, and wrote the manuscript. IP participated to the animal studies, performed flow cytometry analyses and ELISPOT experiments. CT performed immunoblot analyses. KC performed ELISA quantification. RB provided funding and scientific input on IVIg, and revised the manuscript. FC provided funding, conceived and designed the study, analyzed the data, and wrote the manuscript. All authors read and approved the final version of the manuscript.

## Supplementary Material

Additional file 1: Table S1 Antibodies used. Click here for file
