# Supplementary material for: IVIg protects the 3xTg-AD mouse model of Alzheimer’s disease from memory deficit and Aβ pathology
Source: J Neuroinflammation. 2014 Mar 22;11:54. doi: 10.1186/1742-2094-11-54 (PMC3997966; doi:10.1186/1742-2094-11-54)
Supplement: Additional file 1: Table S1 — Antibodies used. [file 1742-2094-11-54-S1.docx]

Supplement TABLE 1: Antibodies used

| **WESTERN BLOT ANTIBODIES** | | | | |
| --- | --- | --- | --- | --- |
| **Antibody** | **Clone** | **Specificity** | **Host** | **Source** |
| ADAM-10 |  | ADAM-10 a.a. 732-748 | Rabbit | EMD Millipore (Billerica, MA, U.S.A.) |
| APP/Aβ | 6E10 | APP a.a. 1-16 | Mouse | Covance, Inc. (Princeton, NJ, U.S.A.) |
| β-actin |  | β-actin | Mouse | Applied Biological Materials (Richmond, BC, Canada) |
| Fractalkine |  | Mouse fractalkine (CX3CL1) | Rabbit | eBioscience, Inc. (San Diego, CA, U.S.A.) |
| CX3CR1 |  | Human/mouse CX3CR1 | Goat | R&D Systems, Inc. (Minneapolis, MN, U.S.A.) |
| Drebrin | Mx823 | c-term peptide (a.a.632-649) | Mouse | Progen Biotechnik GmbH (Heidelberg, Germany) |
| Dynamin 1 |  | Dynamin 1 | Rabbit | Thermo Scientific (Rockford, IL, U.S.A.) |
| GFAP | GA-5 | Glial fibrillary acidic protein | Mouse | Sigma-Aldrich (St.Louis, MO, U.S.A. |
| IgG |  | Human Fcγ specific | Goat | Jackson ImmunoResearch Laboratories Inc. (West Grove, PA, U.S.A.) |
| NF-κb |  | C-term NF-κb p65 | Rabbit | Santa Cruz Biotechnology Inc. (Santa Cruz, CA, U.S.A.) |
| PAK 1/2/3 |  | p21-activated kinase1/PAK2/PAK3 | Rabbit | Cell Signaling Technology (Whitby, ON, Canada) |
| PSD95 | K28/43 | Postsynaptic Density Protein 95 | Mouse | Antibodies Incorporated (Davis, CA, U.S.A) |
| Septin 3 |  | SEPT3 | Rabbit | Novus Biologicals (Oakville, ON, Canada) |
| Snap 25 | SM1 81 | Synaptosomal-associated Protein 25 | Mouse | Covance, Inc. |
| Synaptophysin |  | Synaptophysin | Mouse | EMD Millipore |
| Tau (human) | Tau13 | Total human tau | Mouse | Covance, Inc. |
| Tau (phospho) | AT 270 | Tau, phosphorylated at the Thr-181 | Mouse | Pierce Endogen Inc. (Rockford, IL, U.S.A.) |
| Tau (phospho) | PHF-1 | Tau, phosphorylated at the Ser-396 and Ser-404 | Mouse | Generous gift from Peter Davies |
| Vilip-1 |  | Visinin Like-1 a.a. 1-167 | Rabbit | GeneTex Inc. (Irvine, CA, U.S.A.) |
| YKL-40 |  | Also called CHI3L1 | Rabbit | LifeSpan BioSciences Inc. (Seattle, WA, U.S.A.) |
| **FACS ANTIBODIES** | | | | |
| **Antibody** | **Clone** | **Specificity** | **Host** | **Source** |
| B220 | RA3-6B2 | Also called CD45R | Rat | eBioscience, Inc. |
| CD3 | 17A2 | Cluster of differentiation 3 | Rat | BD Bioscience (Mississauga, On, Canada) |
| CD4 | GK1.5 | Cluster of differentiation 4 | Rat | eBioscience, Inc. |
| CD45 | 30-F11 | Cluster of differentiation 45 | Rat | eBioscience, Inc. |
| CD8b | eBioH35-17.2 | Cluster of differentiation 8b | Rat | eBioscience, Inc. |
| CX3CR1 |  | Human/mouse CX3CR1 | Goat | R&D Systems, Inc. |
| Gr1 | RB6-8C5 | Also called Ly-6G | Rat | eBioscience, Inc. |
| F4/80 | BM8 |  | Rat | eBioscience, Inc. |
